# Current State of CAR T-Cell Therapy in Chronic Lymphocytic Leukemia

**DOI:** 10.3390/ijms22115536

**Published:** 2021-05-24

**Authors:** Veronika Mancikova, Michal Smida

**Affiliations:** Central European Institute of Technology (CEITEC), Masaryk University, 62500 Brno, Czech Republic

**Keywords:** chimeric antigen receptor, immunotherapy, chronic lymphocytic leukemia, CD19

## Abstract

Chimeric antigen receptor (CAR) T-cell therapy has already achieved remarkable remissions in some difficult-to-treat patients with B-cell malignancies. Although the clinical experience in chronic lymphocytic leukemia (CLL) patients is limited, the proportion of remissions reached in this disease is clearly the lowest from the spectrum of B-cell tumors. In this review, we discuss the antigenic targets exploited in CLL CAR-T therapy, the determinants of favorable responses, as well as the mechanisms of treatment failure specific to this disease.

## 1. Introduction

Chronic lymphocytic leukemia (CLL) is a lymphoproliferative disease of neoplastic B cells, which accumulate in the blood, bone marrow, lymph nodes, and spleen [1]. CLL is the most common adult leukemia in Western countries. It is a disease of the elderly, with a median age at diagnosis of 72 years. As such, CLL clinical management is oftentimes challenging and highly individualized based on the age of patients, their comorbidities, and the biological features of CLL cells [2].

Hematopoietic stem cell transplantation (HSCT) is still the only possibly curative therapeutic option, but it is rarely indicated due to its high procedure-related risk [3]. Its application is reserved for a specific subgroup of high-risk CLL patients, defined by clinical and/or genetic resistance (*TP53* abnormalities) to treatment with chemoimmunotherapy and unresponsiveness to pathway inhibitors [4]. In the remaining cases, other treatment options are applied instead, ranging from the watch-and-wait approach in the case of early-stage asymptomatic CLL to chemoimmunotherapy or targeted therapy with specific B cell pathway inhibitors, capable of inducing prolonged remission in patients with more advanced disease [1]. In recent decades, chimeric antigen receptor (CAR) T-cell immunotherapy emerged as another exciting treatment modality for high-risk relapsed/refractory (r/r) CLL patients with resistance to other treatment modalities, complex cytogenetics, and/or *TP53* abnormalities [5], who would otherwise have dismal prognoses. Generally, a lymphodepleting regimen is applied prior to CAR T-cell infusion to potentiate the clinical effect [6]. CAR T cells are T lymphocytes that have been genetically modified to express CAR on their surface. CARs are artificially engineered fusion proteins that incorporate antigen-recognition moieties and T-cell activation domains in a modular manner. They exploit antibody-derived single-chain variable fragments (scFvs) for targeting surface molecules in their native conformation [7]. Thus, CARs are capable of major histocompatibility complex-independent tumor cells’ recognition, extending the T-cell specificity to any target of choice, as long as there is an antibody available for it.

The T-cell response intensity can be fine-tuned by incorporating different activation domains in the CAR design, making this system highly adjustable. At first, only the CD3ζ domain was fused in tandem with the antigen-recognition module. However, these so-called first-generation CARs showed only a limited clinical effect [8]. The addition of a costimulatory signaling domain (e.g., CD28 or 4–1BB) produced the second generation of CARs, which showed significantly improved efficacy [9] and remain the most used design nowadays. Coupling of two costimulatory domains into the receptor sequence distinguishes the third generation CARs, whereas the fourth generation CARs are enhanced even further, e.g., with transgenes for cytokine secretion or other modifications [10].

Although this immunotherapy proved to be a true breakthrough in cases of certain B-cell malignancies [11,12], where it has already been approved for application outside of clinical trials, the benefit of CAR T cells in CLL has not been as clear [13]. In the present review, we discuss the CLL target antigens together with clinical and pre-clinical data supporting their selection, the determinants of clinical responses in CLL, as well as the mechanisms of treatment resistance specific to the disease.

## 2. Target Antigen

The CAR target should ideally be immunogenic, completely tumor-specific, and highly expressed on the surface of all tumor cells [14]. In this regard, CLL cells are characterized by expression of candidate surface antigens CD19, CD20, and CD23 [15], and either κ or λ immunoglobulin light chain [16]. Some of these markers have already become relevant antigenic targets for CAR T-cell therapy in clinical trials (Table 1), while other surface molecules are still only in the preclinical stages of development.

### 2.1. CD19

CD19, encoded by the CD19 gene located on the short arm of chromosome 16, is a transmembrane protein specifically expressed on the surface of all B-lineage cells, with the exception of plasma cells. Likely due to its role in B-cell receptor signaling [33], CD19 expression is rarely lost upon B-cell neoplastic transformation [34,35]. Consequently, it is still the most preferred antigenic target in CAR T-cell clinical trials of CLL patients.

Over 100 CLL patients have been treated with anti-CD19 CAR T cells to date (Table 1). Apart from one study, which enrolled cases with only a partial response to first-line chemoimmunotherapy [25], the remaining patients were heavily pre-treated, and the majority of them relapsed and/or were refractory to conventional therapy regimens. Nine patients experienced a relapse after HSCT [20,23]. In consequence, most of the patients received autologously derived CAR T cells, whereas those recurring after HSCT had their infusion products prepared from donors’ lymphocytes. Of note, the most recent study provided promising evidence that the application of off-the-shelf allogeneic CAR natural killer (NK) cells produced from cord blood is also feasible in CLL, without the need for HLA matching [29].

Notably, the study protocols of some recent trials have evaluated several therapy variations since their first use in CLL in 2011 [5]. Currently, several groups have tested the use of a controlled ratio of 1:1 CD4^+^:CD8^+^ cells in the final infusion product [24,27,28], which can hypothetically improve the clinical efficacy. Additionally, the use of a humanized anti-CD19 scFv in the CAR design [26] as opposed to the mice-derived sequence still used most frequently, may potentially help to minimize the immune reaction of the recipient to the treatment, thus increasing the possibility of a successful engraftment. Recently, a large study concluded that a high dose of 5 × 10^8^ CAR T cells may be more effective at inducing CR in advanced CLL [30].

The overall response rate (ORR; sum of CRs and PRs) varies widely among the studies published so far, and it is challenging to draw conclusions regarding the efficacy. However, studies with the lowest ORR (Table 1 [17,20,23]) commonly lack the application of lymphodepleting chemotherapy prior to CAR T-cell infusion in the study protocol. This common feature once more underlines the importance of proper conditioning of the recipient, which strongly impacts the treatment efficacy. It is thought that this is due to a combination of a decrease in residual tumor mass and of the number of regulatory cells, which may trigger a reaction against the infused CAR T cells. Additionally, a slightly better ORR was achieved in trials incorporating the 4–1BB costimulatory domain in the CAR design (66% vs. 56%), which agrees with the preclinical data showing enhanced antileukemic efficacy and survival of this specific design [9]. Finally, long-term ibrutinib therapy prior to T-cell collection was shown to have a positive impact on CAR T-cell expansion, and thus treatment efficacy [36].

The average rate of CR induction in anti-CD19 studies listed in Table 1 is 32% (ranging from 0% to 60%). Of note, many of these remissions were durable [18,19,21,22,23,29,30,37]. Yet, considering the remissions induced by anti-CD19 CAR T cells in other B-cell-derived malignancies (68% to 93% in patients with acute B-lymphoblastic leukemia (ALL) [11,38], and 64 to 86% in B-cell lymphomas [12,39,40]), the efficacy of the therapy is far below expectations in the CLL scenario. The therapy failure was mostly caused by insufficient T-cell expansion and persistence upon infusion [22,23,24], and was also more frequent among patients with a bulky, aggressive nodal disease [24]. Additionally, although patients with recurrent/persistent disease after CAR T-cell infusions generally remained CD19^+^ [20], a significant portion of patients experienced a disease relapse through antigen-negative escape mechanisms (e.g., a CD19 dim Richter’s transformation [22] or a CD19^−^ relapse [24]).

### 2.2. CD20

B-lymphocyte antigen CD20 is expressed on the surface of all B cells beginning at the late pro-B-cell stage and progressively increasing in concentration until maturity. Interestingly, the exact biologic function of CD20 is unknown, since its involvement in BCR signaling and its alleged function as a calcium channel remain controversial [41].

Nevertheless, CD20 is one of the oldest CLL immunotherapeutic targets. It has been very successfully exploited in CLL treatments with monoclonal antibodies, such as rituximab, ofatumumab, and obinutuzumab [42], although its prolonged therapeutic targeting was associated with downregulation of its expression [43]. Still, encouraging data exist from pivotal anti-CD20 CAR T-cell clinical trials in B-cell lymphomas [44,45], and several anti-CD20 CAR T-cell clinical trials are currently recruiting r/r CLL patients (e.g., NCT03277729). Additionally, three CLL patients were recently enrolled in a clinical trial treating patients with various B-cell malignancies using bispecific anti-CD20/anti-CD19 CAR T-cells (Table 1). Two of the CLL patients experienced durable ongoing remissions, and the third patient reached a partial response [31]. If these encouraging results are replicated in more patients, the latter approach may represent a tool to avoid the emergence of a CD19^−−^ recurrence. Recurrent/persistent disease recorded in this trial was not due to an antigen-negative escape (neither CD19 nor CD20), but rather to inefficient T-cell engraftment.

### 2.3. κ Immunoglobulin Light Chain

Targeting of the pan-B cell markers CD19 and CD20 by CAR T cells generally leads to the long-term impairment of humoral immunity due to B-cell aplasia, induced by a successful treatment. Therefore, alternative targets were investigated [46]. In this regard, B lymphocytes express surface monoclonal immunoglobulins with either κ or λ light chains. Since κ/λ expression is clonally restricted, the malignant CLL cells in a given patient express either κ or λ light chain [47]. Thus, CARs targeting the light chain expressed by the tumor should spare normal B cells expressing the reciprocal light chain. Preclinical in vitro and in vivo data using CLL cell lines as well as primary CLL cells showed a potent cytotoxic effect of anti-κ CAR T-cells against Igκ^+^ tumors [48]. This antigenic target was further explored in a phase 1 clinical trial including two CLL patients (Table 1). A stable disease was the best response achieved in CLL in this trial [32]. Notably, there was no lymphodepletion applied in these patients prior to anti-κ CAR T-cell infusion, which could hamper the efficacy of the therapy and thus partially explains the suboptimal outcome.

### 2.4. Antigens in Preclinical Development

The receptor tyrosine kinase-like orphan receptor 1 (ROR1) was identified as a highly expressed gene in CLL, but not in normal B cells or in any major adult tissue apart from low levels in adipose tissue, pancreas, lung, and at early stage B-cell development. Thus, its targeting by CAR should lead to a tumor-specific elimination. In vitro anti-ROR1 CAR T cells exert cytotoxicity towards primary CLL cells [48], and the treatment is well-tolerated in vivo in nonhuman primates [49]. Consequently, a phase 1 clinical trial (NCT02706392) is currently recruiting refractory CLL patients to evaluate the efficacy of anti-ROR1 CAR T cells in this disease.

CD37 is yet another transmembrane molecule studied as a possible target for CARs in CLL. Its expression is restricted to lymphoid tissues, in particular to mature B-cells, with low levels of expression on plasma cells and dendritic cells, and it is maintained even after neoplastic transformation. In vitro anti-CD37 CAR T cells were activated in the presence of primary CLL cells, and in vivo, they exhibited antitumor cytotoxic activity against other B-cell malignancies. Additionally, bispecific anti-CD37/anti-CD19 CAR T cells were already successfully prepared for preclinical research purposes [50].

Other alternative antigens were searched for to expand the portfolio of CLL targets, which would be specific and sustained in their expression. Particular interest lies in the identification of a target unique for CLL cells that is absent in normal healthy B cells. The Fcµ receptor seems to be one such candidate with consistently high CLL expression and rather low presence on normal B cells. FcµR-specific CAR T cells were shown to successfully eliminate leukemic cells, whereas healthy B cells were spared, both in vitro and in vivo [51]. Targeting this antigen would thus avoid the long-term B-cell aplasia commonly associated with current CAR T-cell targets.

## 3. Determinants of Clinical Response and Treatment Resistance

Considering the results from all clinical trials, approximately only every third CLL patient will reach a complete remission with CAR T-cell therapy. Thus, it would be highly desirable to identify markers that can predict the success of this technically and economically demanding therapy in the CLL scenario. Determinants intrinsic to the infusion product as well as disease-specific characteristics may affect the clinical efficacy of this therapy. Likely due to the limited clinical experience (the largest trials only enrolled a few tens of patients), only a handful of studies are addressing this need so far; further research is urgently needed.

A central driving feature of CLL pathogenesis is early immune deficiency, which promotes tumor expansion and evasion of immune surveillance [52]. Clearly, this defect affecting T lymphocytes, which can be further aggravated by therapy (especially chemotherapy), may impair the expansion and activity of CAR T cells. Interestingly, the tumor microenvironment in CLL also differs from the environment found in ALL patients, likely contributing to the differential efficiency of CAR T-cell therapy in these two entities. It was shown that CLL cells have an impact on T cells, leading to severe skewing of T-cell repertoire, particularly in effector memory T cells, and inducing changes in T-cell gene expression profiles [53]. An analysis of global gene expression in purified CD4^+^ and CD8^+^ T cells from CLL patients revealed profound changes in the expression of genes mainly involved in cell differentiation, actin cytoskeletal reorganization, vesicle trafficking, and cytotoxicity pathways [54]. Consequently, T cells derived from CLL patients showed impaired formation of immune synapse with leukemia cells and defective recruitment of key regulatory proteins to the synapse. However, this defect could be partially reversed by immunomodulatory drugs such as lenalidomide [55].

T cells isolated from CLL patients also showed dysregulated expression of immune checkpoint molecules programmed cell death 1 (PD-1) and cytotoxic T-lymphocyte antigen 4 (CTLA-4) and more activated phenotype [56]. Higher proportions of antigen-experienced T cells were observed in CLL patients, with high PD-1 expression. Moreover, higher numbers of both CD4^+^ and CD8^+^ cells expressing PD-1 were found; higher numbers of T cells with intracellular CTLA-4 and high numbers of proliferating and activated CD4^+^ and CD8^+^ cells. These alterations were more pronounced in the patients with active disease. Another study demonstrated that CLL-derived T cells display features of cell exhaustion with overexpression of exhaustion markers CD244, CD160, and PD-1 [57]. Other markers of exhaustion, such as CTLA-4, TIM3, or LAG3, were found to be normal. On the contrary, these T cells retained the capacity for cytokine production as they showed normal IL-2 production, increased production of IFN-γ and TNFα, and a higher TBET expression. CD8^+^ T cells derived from CLL patients demonstrated defects in granzyme granule relocalization into the immune synapse. Consequently, they exerted defects in proliferation and target-cell killing.

These findings suggest that strategies combining anti-CD19 CAR T cells with immune checkpoint inhibitors or other methods to stimulate T-cell recognition of tumor cells would be appropriate. Interestingly, it was found that five or more cycles of ibrutinib, a BTK inhibitor often used in CLL therapy, especially for the management of adverse prognosis patients [58], caused a decreased expression of the immunosuppressive molecule PD-1 on T cells. Moreover, ibrutinib therapy improved the expansion of anti-CD19 CAR T cells in vivo [36]. These data likely explain the encouraging results observed in several clinical trials combining CAR T-cell therapy with ibrutinib [26,28].

The leading cause of CAR T-cell treatment failure is the inefficient engraftment of the infusion product. The largest study so far, gathering 41 CLL patients for the analyses, identified the mechanistically relevant T-cell subpopulation responsible for mediating tumor control, and underscored the need for minimizing culture time, using alternative cytokines or performing metabolic engineering or other approaches, such as IL-6/STAT3-pathway modulation, in the production process in order to maintain a less-differentiated phenotype of T cells in the infusion product. In detail, an increased proportion of memory-like CD45RO^−^CD27^+^CD8^+^ T cells was found to be associated with favorable responses to CAR-T therapy. The effectiveness of CAR T-cell therapy for CLL was improved by treating patients with cells enriched in CD27^+^PD-1^−^CD8^+^ lymphocytes that expressed high IL-6 receptor levels [13]. The presence of this CAR T-cell subpopulation in the infusion product may thus be used as a marker for predicting efficient therapeutic responses. Likewise, upregulation of genes involved in T-cell exhaustion, activation, glycolysis, and apoptosis in the patient’s T cells might serve as exclusion criteria in determining likely non-responding cases.

The locations of CAR vector integration acceptor sites in the T-cell genome seem to play a role in the clinical outcome [59,60]. Firstly, a single CLL case of CAR-triggered insertional mutagenesis, which led to T-cell clonal expansion and eventually to complete remission, was described. In this particular case, the anti-CD19 CAR vector was integrated into the locus of the *TET2* gene, encoding a demethylation enzyme, sharply reducing the activity of the disrupted protein. This completely stochastic integration event led to the rapid expansion and domination of a single CAR T-cell clone, exhibiting a less-differentiated central memory phenotype in the patient [59]. This result was promptly validated in a study where the locations of CAR vector integration sites were correlated with clinical outcomes in 29 CLL and 11 ALL patients. Several other hotspot sites were identified, which, when disrupted by the transgene insertion, led to a preferential expansion and long-term persistence of a specific clone. Additionally, a multivariate model based on integration site distributions in the pre-infusion products capable of forecasting response in CLL was developed in the mentioned study [60].

Finally, data from clinical studies have so far failed to identify any clear patient- or disease-specific factors predicting which subjects will respond best to CAR T-cell immunotherapy [13,22]. No association between the response and patient age, previous therapies, disease stage, IGHV mutation status, or CD19 surface level has been observed. However, patients with predominantly nodal disease seem to beneficiate less from this particular treatment [24]. This may be partially remedied by concurrent ibrutinib treatment, which is known to cause an efflux of tumor cells from the tissue compartments into the blood [61]. Additionally, some preclinical evidence exists showing that *TP53*-mutated CLL gives rise to early disease onset, high tumor burden, and inefficient T-cell engraftment in a mouse model, leading to inferior performance of anti-CD19 CAR T cells [62]. When we closely inspected patient lists from anti-CD19 clinical trials (Table 1), only 3 of 20 cases unequivocally bearing del17p (*TP53* genetic locus) reached complete remissions.

It has been calculated that up to 20% of disease relapses in B-cell ALL and lymphomas treated with anti-CD19 CAR T cells arise due to tumor escape characterized by the lack of CD19 expression on the surface of neoplastic cells [63,64]. Even though the clinical experience is limited in CLL, such relapses have already been recorded [22,24], and they will likely affect more CLL patients in the future. Some of the mechanisms driving these relapses in other B-cell malignancies are already known. CD19 gene mutations [65], alternative splicing of CD19 mRNA [66], and cell lineage switch [67,68] are among the mechanisms already described to drive antigen-negative escape in CD19^−−^ relapses of ALL. It remains to be determined whether these mechanisms are present also in the CLL setting (which is highly likely), and what other alterations occur in relapsed CLL patients. Of note, epigenetic mechanisms may presumably play a role in CAR T-cell-triggered CD19 regulation, as CD19 promoter hypermethylation causing a lack of CD19 surface expression was recently described in a preclinical relapse model of CLL (manuscript under revision).

## 4. CAR T-Cell-Induced Toxicities in CLL

CAR T-cell therapy is classically associated with cytokine release syndrome (CRS), which reflects the biological mechanism of action of this treatment [69]. In the case of anti-CD19 CAR T cells, CRS is initiated by the activation and proliferation of CAR T-cells after CD19^+^ target cell recognition, leading to production of inflammatory cytokines (such as IL-6, IFN-γ, IL-10, etc. [70]), ultimately causing a plethora of symptoms such as fever, vomiting, seizures, and even organ failure. CRS has oftentimes been reported in CLL clinical trials [5,17,18,19,21,22,23,24,25,26,27,28,30]. However, even the more severe cases were generally reversible when treated with tocilizumab (antibody against IL-6 receptor) and corticosteroids. It is difficult to draw conclusions regarding the incidence of this complication since the available studies only describe clinical experience with a handful of patients. However, the largest series published so far found a significant positive correlation between the percentage of leukemic B cells in marrow before therapy and the incidence of CRS. Likewise, patients affected by CRS had higher peak numbers of CAR T cells in the blood after infusion, accompanied by higher peak values of several cytokines [24]. Finally, there are indications that concomitant CAR T-cell and ibrutinib therapy may decrease the incidence of severe CRS [26,27,71], presumably due to lower levels of inflammatory cytokines found in the patients receiving this combinatory treatment [71].

Although CRS is an expected toxicity of CAR T-cell immunotherapy, unexpected neurological complications (such as cerebral edema) have also been reported in CLL clinical trials, some of which were severe [71] or even fatal [24]. Nevertheless, neither the moment of onset nor the severity of the described toxicities (CRS and/or neurotoxicities) seem to differ from other hematological diseases; therefore, no CLL-specific instructions are currently used in clinical trials [72]. In this regard, the use of the off-the-shelf allogeneic CAR NK-cells, while inducing clinical response, was not associated with the development of any major toxic effects [29].

## 5. Conclusions and Future Perspectives

CAR T-cell therapy is an appealing treatment modality in CLL as it induces durable remissions in patients with unfavorable prognoses. Additionally, recent advances have provided some crucial data, which may help to potentiate the clinical effect even further. Concurrent use of ibrutinib, inducing lymphocytosis as well as partially reversing T-cell exhausted phenotype in the CLL patients, seems especially promising. Locus-specific insertion of the transgenic vector in the genome of recipient T cells with systems such as CRISPR/Cas9 may further improve the efficacy of the treatment. The generation of allogeneic CAR T cells may help to overcome the issue of low-quality or non-functional T cells that are often leukapheresed from heavily pretreated CLL patients. On the contrary, the use of selected T-cell subpopulations, defined CAR T-cell subtype compositions, or combinations of CAR T cells with immune checkpoint inhibitors (or other yet to be determined inhibitors) may further boost the therapeutic response in CLL patients. Bispecific CARs targeting two antigens on the surface of the cancer cell may alleviate the emergence of an antigen-negative relapse. Finally, it will be essential to identify suitable biomarkers dissecting responders from non-responders in order to tailor treatment and to avoid unsuccessful infusions with this costly and complex cellular therapy.

## Figures and Tables

**Table 1:** CAR T-cell clinical trials carried out in CLL to date. r/r, relapsed/refractory; *, relapsed after hematopoietic stem cell transplantation (HSCT); ORR, overall response rate; CR, complete response; PR, partial response; SD, stable disease; PD, progressive disease; NR, no response; NK, natural killer.

Study	Target	Participants	Infusion Product	Comment	Clinical Efficacy	% ORR
[5]	CD19	1 r/r	Autologous bulk T-cells; 2nd generation with 4–1BB		1× CR	100
[17]	CD19	8 r/r	Autologous bulk T-cells; 2nd generation with CD28	No conditioning in 3 patients	3× SD, 4× PD, 1× not evaluable	0
[18]	CD19	3 r/r	Autologous bulk T-cells; 2nd generation with 4–1BB		2× CR, 1× PR	100
[19]	CD19	4 r/r	Autologous bulk T-cells; 2nd generation with CD28	Combined with IL-2	1× CR, 2× PR, 1× SD	75
[20]	CD19	4 r/r*	Allogeneic bulk T-cells; 2nd generation with CD28	HSCT donor-derived CAR T-cells, no conditioning	1× PR, 1× SD, 2× PD	25
[21]	CD19	5 r/r	Autologous bulk T-cells; 2nd generation with CD28		3× CR, 2× PR	100
[22]	CD19	14 r/r	Autologous bulk T-cells; 2nd generation with 4–1BB		4× CR, 4× PR, 6× NR	57
[23]	CD19	5 r/r*	Allogeneic bulk T-cells; 2nd generation with CD28	HSCT donor-derived CAR T cells, no conditioning	1× CR, 1× PR, 1× SD, 2× PD	40
[24]	CD19	24 r/r	Autologous bulk T-cells; 2nd generation with 4–1BB	1:1 CD 4 +: CD8+ ratio for infusion	4× CR, 13× PR, 1× SD, 5× PD, 1× not evaluable	71
[25]	CD19	8 PR after 1^st^ chemoimmunotherapy	Autologous bulk T-cells; 2nd generation with CD28		2× CR, 3× SD, 3× PD	75
[26]	CD19	19 r/r	Autologous bulk T-cells; 2nd generation with 4–1BB	Humanized anti-CD19 scFv, concurrent ibrutinib	10× CR, 1× PD, 8× not evaluable	53
[27]	CD19	10 r/r	Autologous bulk T-cells; 2nd generation with 4–1BB	1:1 CD 4 +: CD8+ ratio for infusion	4×CR, 2×PR, 2×NR, 2× not evaluable	60
[28]	CD19	19 r/r	Autologous bulk T-cells; 2nd generation with 4–1BB	1:1 CD 4 +:CD8+ ratio for infusion, concurrent ibrutinib	4× CR, 11× PR, 3× NR, 1× not evaluable	79
[29]	CD19	5 r/r	Allogeneic NK-cells 4th generation with CD28, IL-15, and inducible caspase 9	CAR NK cells derived from cord blood	3× CR, 1× PR, 1× NR	80
[30]	CD19	32 r/r	Autologous bulk T-cells; 2nd generation with 4–1BB	Low (5 × 10^7^) or high (5 × 10^8^) dose of CAR T-cells	9× CR, 5×PR, 18× NR	44
[31]	CD19 + CD20	3 r/r	Autologous bulk T-cells; 2nd generation with 4–1BB	Bispecific design	2× CR, 1× PR	100
[32]	Igκ	2 r/r	Autologous bulk T-cells; 2nd generation with CD28		1× SD, 1× PD	0

## References

[1] Hallek M., Cheson B.D., Catovsky D., Caligaris-Cappio F., Dighiero G., Döhner H., Hillmen P., Keating M., Montserrat E., Chiorazzi N. (2018). iwCLL guidelines for diagnosis, indications for treatment, response assessment, and supportive management of CLL. Blood.

[2] Eichhorst B., Robak T., Montserrat E., Ghia P., Hillmen P., Hallek M., Buske C. (2015). Chronic lymphocytic leukaemia: ESMO Clinical Practice Guidelines for diagnosis, treatment and follow-up. Ann. Oncol..

[3] Dreger P., Schetelig J., Andersen N.S., Corradini P., Van Gelder M., Gribben J.G., Kimby E., Michallet M., Moreno C., Stilgenbauer S. (2014). Managing high-risk CLL during transition to a new treatment era: Stem cell transplantation or novel agents?. Blood.

[4] Dreger P., Ghia P., Schetelig J., Van Gelder M., Kimby E., Michallet M., Moreno C., Robak T., Stilgenbauer S., Montserrat E. (2018). High-risk chronic lymphocytic leukemia in the era of pathway inhibitors: Integrating molecular and cellular therapies. Blood.

[5] Porter D.L., Levine B.L., Kalos M., Bagg A., June C.H. (2011). Chimeric Antigen Receptor–Modified T Cells in Chronic Lymphoid Leukemia. N. Engl. J. Med..

[6] Bechman N., Maher J. (2021). Lymphodepletion strategies to potentiate adoptive T-cell immunotherapy—What are we doing; where are we going?. Expert Opin. Biol. Ther..

[7] Eshhar Z., Waks T., Bendavid A., Schindler D.G. (2001). Functional expression of chimeric receptor genes in human T cells. J. Immunol. Methods.

[8] Brocker T., Karjalainen K. (1995). Signals through T cell receptor-zeta chain alone are insufficient to prime resting T lymphocytes. J. Exp. Med..

[9] Milone M.C., Fish J.D., Carpenito C., Carroll R.G., Binder G.K., Teachey D., Samanta M., Lakhal M., Gloss B., Danet-Desnoyers G. (2009). Chimeric Receptors Containing CD137 Signal Transduction Domains Mediate Enhanced Survival of T Cells and Increased Antileukemic Efficacy In Vivo. Mol. Ther..

[10] Brentjens R.J., Curran K.J. (2012). Novel cellular therapies for leukemia: CAR-modified T cells targeted to the CD19 antigen. Hematol. Am. Soc. Hematol. Educ. Program.

[11] Maude S.L., Laetsch T.W., Buechner J., Rives S., Boyer M., Bittencourt H., Bader P., Verneris M.R., Stefanski H.E., Myers G.D. (2018). Tisagenlecleucel in Children and Young Adults with B-Cell Lymphoblastic Leukemia. N. Engl. J. Med..

[12] Locke F.L., Ghobadi A., Jacobson C.A., Miklos D.B., Lekakis L.J., Oluwole O., Lin Y., Braunschweig I., Hill B.T., Timmerman J.M. (2019). Long-term safety and activity of axicabtagene ciloleucel in refractory large B-cell lymphoma (ZUMA-1): A single-arm, multicentre, phase 1–2 trial. Lancet Oncol..

[13] Fraietta J.A., Lacey S.F., Orlando E.J., Pruteanu-Malinici I., Gohil M., Lundh S., Boesteanu A.C., Wang Y., O’Connor R.S., Hwang W.-T. (2018). Determinants of response and resistance to CD19 chimeric antigen receptor (CAR) T cell therapy of chronic lymphocytic leukemia. Nat. Med..

[14] Wieczorek A., Uharek L. (2013). Genetically modified T cells for the treatment of malignant disease. Transfus. Med. Hemother..

[15] Hulkkonen J., Vilpo L., Hurme M., Vilpo J. (2002). Surface antigen expression in chronic lymphocytic leukemia: Clustering analysis, interrelationships and effects of chromosomal abnormalities. Leukemia.

[16] Witzig T.E., Li C.-Y., Tefferi A., Katzmann J.A. (1994). Measurement of the Intensity of Cell Surface Antigen Expression in B-Cell Chronic Lymphocytic Leukemia. Am. J. Clin. Pathol..

[17] Brentjens R.J., Rivière I., Park J.H., Davila M.L., Wang X., Stefanski J., Taylor C., Yeh R., Bartido S., Borquez-Ojeda O. (2011). Safety and persistence of adoptively transferred autologous CD19-targeted T cells in patients with relapsed or chemotherapy refractory B-cell leukemias. Blood.

[18] Kalos M., Levine B.L., Porter D.L., Katz S., Grupp S.A., Bagg A., June C.H. (2011). T Cells with Chimeric Antigen Receptors Have Potent Antitumor Effects and Can Establish Memory in Patients with Advanced Leukemia. Sci. Transl. Med..

[19] Kochenderfer J.N., Dudley M.E., Feldman S.A., Wilson W.H., Spaner D.E., Maric I., Stetler-Stevenson M., Phan G.Q., Hughes M.S., Sherry R.M. (2012). B-cell depletion and remissions of malignancy along with cytokine-associated toxicity in a clinical trial of anti-CD19 chimeric-antigen-receptor–transduced T cells. Blood.

[20] Cruz C.R.Y., Micklethwaite K.P., Savoldo B., Ramos C.A., Lam S., Ku S., Diouf O., Liu E., Barrett A.J., Ito S. (2013). Infusion of donor-derived CD19-redirected virus-specific T cells for B-cell malignancies relapsed after allogeneic stem cell transplant: A phase 1 study. Blood.

[21] Kochenderfer J.N., Dudley M.E., Kassim S.H., Somerville R.P., Carpenter R.O., Stetler-Stevenson M., Yang J.C., Phan G.Q., Hughes M.S., Sherry R.M. (2015). Chemotherapy-Refractory Diffuse Large B-Cell Lymphoma and Indolent B-Cell Malignancies Can Be Effectively Treated with Autologous T Cells Expressing an Anti-CD19 Chimeric Antigen Receptor. J. Clin. Oncol..

[22] Porter D.L., Hwang W.-T., Frey N.V., Lacey S.F., Shaw P.A., Loren A.W., Bagg A., Marcucci K.T., Shen A., Gonzalez V. (2015). Chimeric antigen receptor T cells persist and induce sustained remissions in relapsed refractory chronic lymphocytic leukemia. Sci. Transl. Med..

[23] Brudno J.N., Somerville R.P., Shi V., Rose J.J., Halverson D.C., Fowler D.H., Gea-Banacloche J.C., Pavletic S.Z., Hickstein D.D., Lu T.L. (2016). Allogeneic T Cells That Express an Anti-CD19 Chimeric Antigen Receptor Induce Remissions of B-Cell Malignancies That Progress After Allogeneic Hematopoietic Stem-Cell Transplantation Without Causing Graft-Versus-Host Disease. J. Clin. Oncol..

[24] Turtle C.J., Hay K.A., Hanafi L.-A., Li D., Cherian S., Chen X., Wood B., Lozanski A., Byrd J.C., Heimfeld S. (2017). Durable Molecular Remissions in Chronic Lymphocytic Leukemia Treated with CD19-Specific Chimeric Antigen Receptor–Modified T Cells After Failure of Ibrutinib. J. Clin. Oncol..

[25] Geyer M., Rivière I., Sénéchal B., Wang X., Wang Y., Purdon T.J., Hsu M., Devlin S.M., Halton E., Lamanna N. (2018). Autologous CD19-Targeted CAR T Cells in Patients with Residual CLL following Initial Purine Analog-Based Therapy. Mol. Ther..

[26] Gill M.S.I., Vides B.V., Frey N.V., Metzger B.S., O’Brien M., Hexner E., Mato M.A.R., Lacey B.S.F., Melenhorst J.J., Pequignot M.E. (2018). Prospective Clinical Trial of Anti-CD19 CAR T Cells in Combination with Ibrutinib for the Treatment of Chronic Lymphocytic Leukemia Shows a High Response Rate. Blood.

[27] Siddiqi T., Soumerai J.D., Wierda W.G., Dubovsky J.A., Gillenwater H.H., Gong L., Mitchell M.A., Thorpe B.J., Yang L., Dorritie K.A. (2018). Rapid MRD-Negative Responses in Patients with Relapsed/Refractory CLL Treated with Liso-Cel, a CD19-Directed CAR T-Cell Product: Preliminary Results from Transcend CLL 004, a Phase 1/2 Study Including Patients with High-Risk Disease Previously Treated with Ibrutinib. Blood.

[28] Gauthier J., Hirayama A.V., Purushe J., Hay K.A., Lymp J., Li D.H., Yeung C.C.S., Sheih A., Pender B.S., Hawkins R.M. (2020). Feasibility and efficacy of CD19-targeted CAR T cells with concurrent ibrutinib for CLL after ibrutinib failure. Blood.

[29] Liu E., Marin D., Banerjee P., Macapinlac H.A., Thompson P., Basar R., Kerbauy L.N., Overman B., Thall P., Kaplan M. (2020). Use of CAR-Transduced Natural Killer Cells in CD19-Positive Lymphoid Tumors. N. Engl. J. Med..

[30] Frey N.V., Gill S., Hexner E.O., Schuster S., Nasta S., Loren A., Svoboda J., Stadtmauer E., Landsburg D.J., Mato A. (2020). Long-Term Outcomes from a Randomized Dose Optimization Study of Chimeric Antigen Receptor Modified T Cells in Relapsed Chronic Lymphocytic Leukemia. J. Clin. Oncol..

[31] Shah N.N., Johnson B.D., Schneider D., Zhu F., Szabo A., Keever-Taylor C.A., Krueger W., Worden A.A., Kadan M.J., Yim S. (2020). Bispecific anti-CD20, anti-CD19 CAR T cells for relapsed B cell malignancies: A phase 1 dose escalation and expansion trial. Nat. Med..

[32] Ramos C.A., Savoldo B., Torrano V., Ballard B., Zhang H., Dakhova O., Liu E., Carrum G., Kamble R.T., Gee A.P. (2016). Clinical responses with T lymphocytes targeting malignancy-associated κ light chains. J. Clin. Investig..

[33] Sato S., Jansen P.J., Tedder T.F. (1997). CD19 and CD22 expression reciprocally regulates tyrosine phosphorylation of Vav protein during B lymphocyte signaling. Proc. Natl. Acad. Sci. USA.

[34] Scheuermann R.H., Racila E. (1995). CD19 Antigen in Leukemia and Lymphoma Diagnosis and Immunotherapy. Leuk. Lymphoma.

[35] Uckun F., Jaszcz W., Ambrus J., Fauci A., Gajl-Peczalska K., Song C., Wick M.R., Myers D., Waddick K., Ledbetter J. (1988). Detailed studies on expression and function of CD19 surface determinant by using B43 monoclonal antibody and the clinical potential of anti-CD19 immunotoxins. Blood.

[36] Fraietta J.A., Beckwith K.A., Patel P.R., Ruella M., Zheng Z., Barrett D.M., Lacey S.F., Melenhorst J.J., Mcgettigan S.E., Cook D.R. (2016). Ibrutinib enhances chimeric antigen receptor T-cell engraftment and efficacy in leukemia. Blood.

[37] Cappell K.M., Sherry R.M., Yang J.C., Goff S.L., Vanasse D.A., McIntyre L., Rosenberg S.A., Kochenderfer J.N. (2020). Long-Term Follow-Up of Anti-CD19 Chimeric Antigen Receptor T-Cell Therapy. J. Clin. Oncol..

[38] Park J.H., Rivière I., Gonen M., Wang X., Sénéchal B., Curran K.J., Sauter C., Wang Y., Santomasso B., Mead E. (2018). Long-Term Follow-up of CD19 CAR Therapy in Acute Lymphoblastic Leukemia. N. Engl. J. Med..

[39] Schuster S.J., Svoboda J., Chong E.A., Nasta S.D., Mato A.R., Anak Ö., Brogdon J.L., Pruteanu-Malinici I., Bhoj V., Landsburg D. (2017). Chimeric Antigen Receptor T Cells in Refractory B-Cell Lymphomas. N. Engl. J. Med..

[40] Schuster S.J., Bishop M.R., Tam C.S., Waller E.K., Borchmann P., McGuirk J.P., Jäger U., Jaglowski S., Andreadis C., Westin J.R. (2019). Tisagenlecleucel in Adult Relapsed or Refractory Diffuse Large B-Cell Lymphoma. N. Engl. J. Med..

[41] Kozlova V., Ledererova A., Ladungova A., Peschelova H., Janovska P., Slusarczyk A., Domagala J., Kopcil P., Vakulova V., Oppelt J. (2020). CD20 is dispensable for B-cell receptor signaling but is required for proper actin polymerization, adhesion and migration of malignant B cells. PLoS ONE.

[42] Huhn D. (2001). Rituximab therapy of patients with B-cell chronic lymphocytic leukemia. Blood.

[43] Hiraga J., Tomita A., Sugimoto T., Shimada K., Ito M., Nakamura S., Kiyoi H., Kinoshita T., Naoe T. (2009). Down-regulation of CD20 expression in B-cell lymphoma cells after treatment with rituximab-containing combination chemotherapies: Its prevalence and clinical significance. Blood.

[44] Zhang W.-Y., Wang Y., Guo Y.-L., Dai H.-R., Yang Q.-M., Zhang Y.-J., Zhang Y., Chen M.-X., Wang C.-M., Feng K.-C. (2016). Treatment of CD20-directed Chimeric Antigen Receptor-modified T cells in patients with relapsed or refractory B-cell non-Hodgkin lymphoma: An early phase IIa trial report. Signal Transduct. Target. Ther..

[45] Till B.G., Jensen M.C., Wang J., Qian X., Gopal A.K., Maloney D.G., Lindgren C.G., Lin Y., Pagel J.M., Budde L.E. (2012). CD20-specific adoptive immunotherapy for lymphoma using a chimeric antigen receptor with both CD28 and 4-1BB domains: Pilot clinical trial results. Blood.

[46] Vera J., Savoldo B., Vigouroux S., Biagi E., Pule M., Rossig C., Wu J., Heslop H.E., Rooney C.M., Brenner M.K. (2006). T lymphocytes redirected against the κ light chain of human immunoglobulin efficiently kill mature B lymphocyte-derived malignant cells. Blood.

[47] Fialkow P., Najfeld V., Reddy A.L., Singer J., Steinmann L. (1978). Chronic lymphocytic leukæmia: Clonal origin in a committed b-lymphocyte progenitor. Lancet.

[48] Hudecek M., Schmitt T.M., Baskar S., Lupo-Stanghellini M.T., Nishida T., Yamamoto T.N., Bleakley M., Turtle C.J., Chang W.-C., Greisman H.A. (2010). The B-cell tumor–associated antigen ROR1 can be targeted with T cells modified to express a ROR1-specific chimeric antigen receptor. Blood.

[49] Berger C., Sommermeyer D., Hudecek M., Berger M., Balakrishnan A., Paszkiewicz P.J., Kosasih P.L., Rader C., Riddell S.R. (2015). Safety of Targeting ROR1 in Primates with Chimeric Antigen Receptor–Modified T Cells. Cancer Immunol. Res..

[50] Scarfò I., Ormhøj M., Frigault M.J., Castano A.P., Lorrey S., Bouffard A.A., Van Scoyk A., Rodig S.J., Shay A.J., Aster J.C. (2018). Anti-CD37 chimeric antigen receptor T cells are active against B- and T-cell lymphomas. Blood.

[51] Faitschuk E., Hombach A.A., Frenzel L.P., Wendtner C.-M., Abken H. (2016). Chimeric antigen receptor T cells targeting Fc μ receptor selectively eliminate CLL cells while sparing healthy B cells. Blood.

[52] Rossi D., Sozzi E., Puma A., De Paoli L., Rasi S., Spina V., Gozzetti A., Tassi M., Cencini E., Raspadori D. (2009). The prognosis of clinical monoclonal B cell lymphocytosis differs from prognosis of Rai 0 chronic lymphocytic leukaemia and is recapitulated by biological risk factors. Br. J. Haematol..

[53] Tinhofer I., Weiss L., Gassner F., Rubenzer G., Holler C., Greil R. (2009). Difference in the Relative Distribution of CD4+ T-cell Subsets in B-CLL With Mutated and Unmutated Immunoglobulin (Ig) VH Genes. J. Immunother..

[54] Görgün G., Holderried T.A.W., Zahrieh D., Neuberg D., Gribben J.G. (2005). Chronic lymphocytic leukemia cells induce changes in gene expression of CD4 and CD8 T cells. J. Clin. Investig..

[55] Ramsay A.G., Johnson A.J., Lee A.M., Görgün G., Le Dieu R., Blum W., Byrd J.C., Gribben J.G. (2008). Chronic lymphocytic leukemia T cells show impaired immunological synapse formation that can be reversed with an immunomodulating drug. J. Clin. Investig..

[56] Palma M., Gentilcore G., Heimersson K., Mozaffari F., Näsman-Glaser B., Young E., Rosenquist R., Hansson L., Österborg A., Mellstedt H. (2017). T cells in chronic lymphocytic leukemia display dysregulated expression of immune checkpoints and activation markers. Haematologica.

[57] Riches J.C., Davies J.K., McClanahan F., Fatah R., Iqbal S., Agrawal S., Ramsay A.G., Gribben J.G. (2013). T cells from CLL patients exhibit features of T-cell exhaustion but retain capacity for cytokine production. Blood.

[58] Byrd J.C., Furman R.R., Coutre S.E., Flinn I.W., Burger J.A., Blum K., Sharman J.P., Wierda W., Zhao W., Heerema N.A. (2020). Ibrutinib Treatment for First-Line and Relapsed/Refractory Chronic Lymphocytic Leukemia: Final Analysis of the Pivotal Phase Ib/II PCYC-1102 Study. Clin. Cancer Res..

[59] Fraietta J.A., Nobles C.L., Sammons M.A., Lundh S., Carty S.A., Reich T.J., Cogdill A.P., Morrissette J.J.D., DeNizio J.E., Reddy S. (2018). Disruption of TET2 promotes the therapeutic efficacy of CD19-targeted T cells. Nat. Cell Biol..

[60] Nobles C.L., Sherrill-Mix S., Everett J.K., Reddy S., Fraietta J.A., Porter D.L., Frey N., Gill S.I., Grupp S.A., Maude S.L. (2019). CD19-targeting CAR T cell immunotherapy outcomes correlate with genomic modification by vector integration. J. Clin. Investig..

[61] Herman S.E.M., Niemann C.U., Farooqui M., Jones J., Mustafa R.Z., Lipsky A., Saba N.F., Martyr S., Soto S., Valdez J.C. (2014). Ibrutinib-induced lymphocytosis in patients with chronic lymphocytic leukemia: Correlative analyses from a phase II study. Leukemia.

[62] Mancikova V., Peschelova H., Kozlova V., Ledererova A., Ladungova A., Verner J., Loja T., Folber F., Mayer J., Pospisilova S. (2019). Performance of anti-CD19 chimeric antigen receptor T cells in genetically defined classes of chronic lymphocytic leukemia. J. Immunother. Cancer.

[63] Xu X., Sun Q., Liang X., Chen Z., Zhang X., Zhou X., Li M., Tu H., Liu Y., Tu S. (2019). Mechanisms of Relapse After CD19 CAR T-Cell Therapy for Acute Lymphoblastic Leukemia and Its Prevention and Treatment Strategies. Front. Immunol..

[64] Shalabi H., Kraft I.L., Wang H.-W., Yuan C.M., Yates B., Delbrook C., Zimbelman J.D., Giller R., Stetler-Stevenson M., Jaffe E.S. (2018). Sequential loss of tumor surface antigens following chimeric antigen receptor T-cell therapies in diffuse large B-cell lymphoma. Haematologica.

[65] Orlando E.J., Han X., Tribouley C., Wood P.A., Leary R.J., Riester M., Levine J.E., Qayed M., Grupp S.A., Boyer M. (2018). Genetic mechanisms of target antigen loss in CAR19 therapy of acute lymphoblastic leukemia. Nat. Med..

[66] Sotillo E., Barrett D.M., Black K.L., Bagashev A., Oldridge D.A., Wu G., Sussman R.T., LaNauze C., Ruella M., Gazzara M.R. (2015). Convergence of Acquired Mutations and Alternative Splicing of CD19 Enables Resistance to CART-19 Immunotherapy. Cancer Discov..

[67] Gardner R., Wu D., Cherian S., Fang M., Hanafi L.-A., Finney O., Smithers H., Jensen M.C., Riddell S.R., Maloney D.G. (2016). Acquisition of a CD19-negative myeloid phenotype allows immune escape of MLL-rearranged B-ALL from CD19 CAR-T-cell therapy. Blood.

[68] Jacoby E., Nguyen S.M., Fountaine T.J., Welp K., Gryder B., Qin H., Yang Y., Chien C.D., Seif A.E., Lei H. (2016). CD19 CAR immune pressure induces B-precursor acute lymphoblastic leukaemia lineage switch exposing inherent leukaemic plasticity. Nat. Commun..

[69] June C.H., O’Connor R.S., Kawalekar O.U., Ghassemi S., Milone M.C. (2018). CAR T cell immunotherapy for human cancer. Science.

[70] Teachey D.T., Lacey S.F., Shaw P.A., Melenhorst J.J., Maude S.L., Jeffrey F., Pequignot E., Gonzalez V.E., Chen F., Finklestein J. (2016). Identification of Predictive Biomarkers for Cytokine Release Syndrome after Chimeric Antigen Receptor T-cell Therapy for Acute Lymphoblastic Leukemia. Cancer Discov..

[71] Gauthier J., Hirayama A.V., Hay M.K.A., Li D., Lymp J., Sheih A., Purushe J., Pender M.B.S., Hawkins B.R.M., Vakil M.A. (2018). Comparison of Efficacy and Toxicity of CD19-Specific Chimeric Antigen Receptor T-Cells Alone or in Combination with Ibrutinib for Relapsed and/or Refractory CLL. Blood.

[72] Lemal R., Tournilhac O. (2019). State-of-the-art for CAR T-cell therapy for chronic lymphocytic leukemia in 2019. J. Immunother. Cancer.

